# Investigating Nurses’ Views on Care of Mentally Ill Patients with Skin Injuries

**DOI:** 10.3390/ijerph17207610

**Published:** 2020-10-19

**Authors:** Evridiki Kaba, Aikaterini Triantafyllou, Georgia Fasoi, Martha Kelesi, Areti Stavropoulou

**Affiliations:** Department of Nursing, School of Health and Care Sciences, University of West Attica, 12243 Athens, Greece; katerina.mee@hotmail.com (A.T.); gfasoi@uniwa.gr (G.F.); mkel@uniwa.gr (M.K.); astavropoulou@uniwa.gr (A.S.)

**Keywords:** views, mental health nurses, skin injuries, mentally ill patients, qualitative research, grounded theory

## Abstract

Background: Individuals with mental illness are at increased risk of skin injuries. The role of nurses in skin injury prevention and management is crucial and therefore their views on wound care may provide useful information for improving the quality of the care provided. Aim: To investigate nurses’ views on care of mentally ill patients with skin injuries. Method: A qualitative research design based on the principles of grounded theory approach was followed. Unstructured interviews were conducted with seven nurses working in psychiatric wards with frequent skin injuries. Data were analyzed using the constant comparative method of analysis. Results: Two main themes were emerged from data analysis. The first main theme, namely factors affecting the care of patients with skin injuries, included seven categories: (a) shortage of hospital supplies, equipment and services (b) staff shortages, (c) lack of knowledge, (d) nurses’ resistance to change, (e) difficulty in collaborating with patients, (f) patients’ physical conditions and (g) nurses’ attitudes as an obstacle to care. The second main theme, namely nurses’ suggestions for optimizing care, included five categories: (a) need for additional staff, (b) need for increasing hospital supplies and equipment availability, (c) need for training (d) need of changing nurses’ attitudes towards care and (e) need of changing the patients’ approach to collaboration. Conclusion: The care of mentally ill patients with skin injuries is affected by multidimensional factors that have a direct impact on the quality of nurses’ work and patients’ hospitalization. Specific efforts are needed to overcome the obstacles that hinder the care provided and to improve clinical practice.

## 1. Introduction

People with mental illness are at higher risk for physical illness and have significantly lower survival expectancy than the general population [1,2]. Patients in psychiatric wards have an increased risk of skin injuries associated with shear and friction forces. The most common skin injuries are pressure ulcers, diabetic ulcers, vascular ulcers, partial thickness burns, skin damage and wounds caused by self-injury [3]. This is due to the use of psychotropic drugs and their side effects and due to the individuals’ lifestyle. Lack of motivation for life, insufficient mobilization and harmful habits contribute to the weakening of the immune system resulting in wound development and difficult recovery. In addition, other factors that increase the incidence of ulcers in psychiatric hospitals are poor nutrition, as a result of depressive episodes, and involuntary or voluntary self-injury due to depression or self-destructive behavior [2].

Although the role of nurses in skin injury prevention and management is crucial, mental health nurses focus mainly on the symptoms of mental illness, neglecting the cause of these injuries, which results in their inadequate management. In addition, patients with mental illness have difficulties in understanding the purpose of wound management and are unable to comply with treatment, making nurses’ work more difficult [2]. Finally, the above factors in combination with the lack of special training of nurses in wound care lead to complications and delayed healing [4]. 

In recent years, much emphasis has been placed on improving the physical health of people with mental illness [5,6]. Studies on caring of mentally ill patients with skin injuries are relatively few, especially within the Greek context. Research usually approaches the problem by either focusing on ulcers as a comorbid mental health problem [7] or focusing on the impact of ulcers on mental health [8]. For these reasons, investigating nurses’ views on the care of mentally ill patients with skin injuries could provide useful information that could help optimize the care provided, through better understanding of the problem and its related risk factors. 

The aim of this qualitative study was to investigate the views of nurses on the care of mentally ill patients with skin injuries. The objectives of the study were:

(a) to identify the various factors that affect nurses’ views on the phenomenon under investigation;
(b) to explore the problems encountered by nurses who care for mentally ill people with skin injuries in psychiatric hospitals.

Accordingly the research questions were formed as follows:

- How do mental health nurses experience the problems encountered while caring for mentally ill patients with skin injuries?
- What factors affect mental health nurses’ views and attitudes about the care provided to mentally ill people with skin injuries in psychiatric hospitals?
- What factors influence the care delivered by mental health nurses to mentally ill people with skin injuries in psychiatric hospitals?

## 2. Material and Method

### 2.1. Design

A qualitative research design based on the principles of grounded theory (GT) was chosen for the present study. Qualitative approaches are the most appropriate for studying and exploring personal experiences, values and attitudes. In particular, qualitative research has been described in the literature as the most appropriate methodology for evaluating the views, attitudes and experiences of nurses [9]. GT was used to gain an in-depth understanding and interpretation of the phenomenon under investigation as it is considered the ideal method for cases or issues that are being investigated for the first time [10]. Qualitative research methods including GT attract nurses for several reasons, as they help them gain new knowledge and improve their practice in a more meaningful way. Studies using GT methodology can generate new nursing knowledge derived from real interactions with patients as well as by interpreting how participants make sense of their perceptions and actions. GT is considered the appropriate research approach in areas where major gaps in the literature exist, and where new perspectives might be needed [11]. This research approach was chosen because there is not enough information about the phenomenon under study.

A purposive sampling strategy was used, as the respondents were typical cases representing the main categories of the research population. The choice of the sample was intentional and not accidental [12]. The study population consisted of mental health nurses working in psychiatric wards with an increased frequency of skin injuries as these may provide in-depth and comprehensive answers on the subject under investigation. Initially, three participants were selected as the purposive sample as they had the appropriate knowledge and experience in the field under investigation (PUs), and then a theoretical sampling was carried out as defined by the principles of GT [10]. In GT studies, the researcher initially identifies a small number of participants to interview who have experienced the phenomenon under study and are able to talk about their experiences in a clear, expressive and thoughtful way (purposive sampling). As the interviews and the analysis proceed, the codes and categories developed from the first data set guide the theoretical sampling. Depending on the results from the first round of data analysis, the researcher may recruit more participants to interview, people who will confirm or disagree with what the researcher has already found. The process of theoretical sampling stops when data saturation is achieved and when no new information emerges from data analysis [11]. 

The following inclusion criteria to the study were defined:

- Mental health nurses who work in psychiatric wards where there is increased frequency of acute and chronic skin injuries, as their views are the most appropriate to be investigated in relation to the research subject.

Exclusion criteria involved:

- Mental health nurses employed in psychiatric wards with a small frequency of skin injuries or nurses who had managerial roles, were part-time or were agency nursing staff due to their limited contact with patients.

For recruitment purposes, the researcher contacted the nurse managers of the study settings in order to inform them about the aim of study and ask for their support. In parallel, the study was advertised to all potential participants through informal meetings.

### 2.2. Data Collection 

Unstructured interviews were used for data collection purposes. In particular, the unstructured interview is open and does not include predefined questions, but broad topics on which participants are asked to speak or express their opinions on freely and on their own terms [12]. The interviews were conducted in a convenient place and time for the participants. Both place and time were arranged and agreed with the study participants. Criteria for selecting the appropriate location for conducting the interview involved maintenance of privacy and data protection. In addition, reassurance that other people, such as employees or patients, would not able to interfere or interrupt the process of the interview, was guaranteed. A tape recorder was used for data collection purposes. At the same time, field notes were kept by the researcher, in order to capture the non-verbal communication with the participant but also to facilitate the process. In this respect, the researcher can return to important information provided by the participant without interrupting them. Before the commencement of each interview, the researcher provided the participants with the necessary information about the study, such as the aim and the purpose of the research and the participants’ selection criteria. The expected benefits of the research were also mentioned, and issues of confidentiality of the data and the rights of the participants were explored. A signed consent form was obtained from each participant before the interview. 

### 2.3. Data Analysis

The approach used to analyzed the data was based on the grounded theory method. Specifically, the constant comparative method was used in data collection and analysis. Constant comparative analysis involves relating data to ideas, and then ideas to other ideas. This is done through “coding” the data. An ongoing and simultaneous collection and analysis of the data guided further data collection, which continued until “theoretical saturation” achieved, whereby no new or relevant insights seemed to be emerging from the data being collected [13,14]. The constant comparative method together with theoretical sampling constitute the core of qualitative analysis in the grounded theory approach developed by Glaser and Strauss [10]. The method of comparing and contrasting is used for practically all intellectual tasks during analysis: forming categories, assigning the segments to categories, summarizing the content of each category and finding disconfirming cases. The interviews were analyzed word by word, line by line and sentence by sentence. Codes were allocated for each word or phrase and labels were given to emerging themes and then coded. Codes were then sorted into categories and redefined into further categories and themes. Then the main themes, the core categories and other connected categories, properties and relations between categories were identified [10,12,15]. In the present study, data analysis was performed manually, without the use of an analysis software. Although the use of software provides a number of advantages, manual analysis of the data is considered to enable the researcher to become particularly familiar with them, to be able to understand them in depth and to highlight new issues [16].

### 2.4. Ethical Considerations

Before the commencement of the study ethical approval was granted by the Scientific Committee of the involved hospital. The researchers acted in accordance with the Code of Nursing Ethics [17,18,19]. Anonymity, voluntary participation and confidentiality of personal data were strictly followed and observed. It was also stressed to the potential participants that the study results would be used only for research purposes and no identifiable information would be revealed. Any identifiable information was kept separate from the rest of the data. The data were stored under the responsibility of the researcher. Permission for tape recording was also requested. The researchers copied the recordings and were able to provide the participants with a copy of the recorded text upon request. In addition, participants were informed about their right to control the recording. If participants request some parts of the interview to be omitted, these will be deleted from all corresponding files. The participants were also informed about their right to withdraw from the study without any consequences and to refuse to answer any questions they wish and remain in the study. Finally, the researcher stated that no financial obligations occur for the involved hospitals and the participants during the proposed study. 

## 3. Results

Two male and five female mental health nurses who worked in two psychiatric wards of the University Hospital of Attica, Greece, participated in the present study. All participants worked in departments with frequent skin injuries. Demographic characteristics of the participants are presented in Table 1. 

The analysis of the data revealed two main themes, namely (a) factors affecting the care of patients with skin injuries and (b) nurses’ suggestions for optimizing care. Accordingly, seven subthemes were included within the first main theme, labeled as the shortage of hospital supplies, equipment and services, shortage of staff, lack of knowledge, nurses’ resistance to change, difficulties in collaborating with patients, patients’ physical condition and the nurses’ attitudes as an obstacle to care. The second main theme included five subthemes, namely, the need for additional staff, need for increasing hospital supplies and equipment availability, need for training need of changing nurses’ attitudes towards care and (e) need of changing the patients’ approach to collaboration (Table 2). 

### 3.1. Factors Affecting the Care of Patients with Skin Injuries 

#### 3.1.1. The Shortage of Hospital Supplies, Equipment and Services 

Lack of resources was considered by the participants as one of the most important obstacles to wound care. Hospital supplies and equipment appeared to be limited in both quantity and suitability. This is highlighted by the following participant:

*“… what is missing here is the right support material, although nurses have the appropriate knowledge to take care of skin injuries, unfortunately there are no proper beds or mattresses … no suitable material for wound changing … we are not sure about the quality and the appropriateness of the material that we use in cleaning and changing the wounds ”*

(P2)

#### 3.1.2. The Shortage of Staff 

An additional factor that impedes the provision of optimal care is staff shortages. This often restricts the care provided to patients with skin injuries, as priority is given to other tasks. Staff shortages are also related to heavy workload, limited time and nurses’ dissatisfaction with their working environment. Specifically, the participants stated:

*“Nurses are not enough, we have 40 patients to take care of … and only 2 nurses in the afternoon and night shift … shortage of staff is a big problem, because priority is given to other duties and not to wound care.”*

(P4)

*“Preventing skin injuries requires appropriate and timeless care, continuing assessment, to change patients’ positions frequently …, this doesn’t happen … why?... because there is not enough time to do it. ”*

(P5)

#### 3.1.3. The Lack of Knowledge 

Lack of nurses’ knowledge on wound care was reported by the participants as an issue that may affect delivery of care for mentally ill patients with skin injuries. Nurses’ demotivation to update their knowledge was also demonstrated by the participants’ statements.

*“Lack of knowledge is a problem … This is a specialized hospital in psychiatric cases. Most of the nurses are trained only in this … they have no other clinical experience … no knowledge in aspects like wound care ... I don’t blame them, they were never given the opportunity to see something different, but I think it’s not all about the others to give you the opportunity, you have to search for it.”*

(P7)

#### 3.1.4. The Nurses’ Resistance to Change 

Participants viewed that resistance to change and persistence with outdated practices were factors that affected wound care. Despite all the recent advantages in wound care therapies and the progress in wound healing, many nurses find it difficult to apply the most recent protocols and guidelines in their daily practice.

*“Unfortunately, my colleagues—it is typical of a psychiatric hospital—find it difficult to change … to adapt new techniques, to use the new materials … and although we have discussed many times that care should be based in the most recent and best evidence they just ignore it by saying … ‘that’s how I know it, that’s how I’ll do it.”*

(P1)

#### 3.1.5. The Difficulty in Collaborating with Patients 

Many participants reported that collaboration with patients with mental illness is often challenging. Mentally ill patients do not easily accept nursing care and this becomes even more difficult when patients need wound care. Feelings of guilt, anger, shame or fear may impede the prevention of skin injuries. Patients’ inability to maintain hygiene and to comply with treatment may also result in a significant risk of infections.

*“Caring for the mentally ill is in itself is difficult, even more when you have to take care of a wound … they often feel guilty or ashamed about the wound, they do not report any pain or bleeding … risk of wound infection is high … the patients do not keep the pads, nor hygiene rules … usually they don’t cooperate in this.”*

(P4)

*“Managing such a patient is difficult … they easily misinterpret everything, so you have to create a trusting relationship with them … to be sure that what you do is for their own good.”*

(P3)

Even more, aggressive behavior and prolonged stabilization of mentally ill patients can make wound care difficult or even impossible, as this is highlighted in participants statements.

*“A sufficient period of time passes until the patient is emotionally and behaviorally stabilized … this severely affects the development of skin injuries.”*

(P4)

*“Very often a patient attacks you during the dressing change, because he is in pain … he can’t realize what is going on … he is trying to defend himself … of course that affects your work …”*

(P3)

#### 3.1.6. The Patients’ Physical Condition 

An additional factor that affects nurses care is the patients’ impaired health status. Τhe co-occurrence of multiple chronic or acute diseases as well as patients’ life style may hinder wound care and healing.

*“Many patients are suffering from other diseases as well … diabetes, obesity and these do not help at all … they often referred to other hospitals for treatment … when they return to us the skin ulcers are worse … we have to start from the beginning…”*

(P1)

*“These patients have unique lifestyles, often harmful that add to the development of pressure ulcers, prolonged bed rest, reduced mobility, smoking, malnutrition, obesity, these destruct care and wound healing.”*

(P5)

#### 3.1.7. The Nurses’ Attitudes as an Obstacle to Care 

Participants stated that often nurses’ attitudes, views and feelings towards caring for mentally ill patients with skin injuries may affect the provision of care. The patients’ age, nurses’ inability to promote wound healing and psychological exhaustion were raised in nurses’ statements.

*“After so many years of practice, I have become emotionally detached especially with the elderly … I say OK, they had their life they may have children or grandchildren … it is not the same as seeing a young person in this situation.”*

(P3)

*“At the beginning (of my career) when a patient was not doing well, I was upset then I realized that I cannot do much … the patient is already bedridden, his condition is bad and it does not matter so much what we will do … ”*.

(P4)

Feelings of frustration, anxiety, lack of patience, anger, lack of motivation and energy and compassion fatigue may affect the provision of care.

*“Caring for these patients creates a sense of failure, a sense of frustration, a sense that we can’t offer enough … at the beginning you may think you can change everything, after two years in such a stressful environment you feel tired.”*

(P7)

*“I often see my colleagues get angry, to treat patients abruptly, I see colleagues who are apathetic after so many years of work, have lost their interest and motivation. Therefore, they do not offer what they could offer.”*

(P5)

### 3.2. Nurses’ Suggestions for Optimizing Care 

#### 3.2.1. Need for Additional Staff 

Optimal care was associated with improving the nurse–patient ratio. The need to recruit registered nurses was highlighted by the participants. They further referred to the necessity of recruitment of nurses specialized in wound care.

*“… We need more nursing staff, we have asked for it countless times … more nurses are needed in each shift.”*

(P2)

*“In fact we need specialized nurses … if in each shift there are two nurses who change the wounds, the next day different nurses do so … there is no continuity of care. Different people, different practices, every person stands with his own knowledge, which in many cases is neither sufficient nor even substantiated … we need staff, specialized staff.”*

(P6)

#### 3.2.2. Need for Increasing Hospital Supplies and Equipment Availability 

The majority of participants highlighted the need for increasing hospital supplies and equipment as a necessary action to improve care.

*“The most important step is to increase the supplies of the materials needed for wound care. The management must take the appropriate action, to ask for increased supplies, sufficient quantities of proper material … it is not possible to provide care without proper dressings …”*

(P4)

#### 3.2.3. Need for Training

According to participants, the nurses’ lack of knowledge on wound care is an inhibiting factor to healing. The need for lifelong training was stressed by several participants.

*“I have already asked the management to organize seminars, to invite specialists to give a lecture, to do a demonstrations on how to use the dressings, how to clean the wound … I believe that knowledge, continuous education, seminars will help the nurses to understand their responsibilities, to be accountable of the care rendered.. to realize that this is their job, not the surgeons’ job.”*

(P3)

#### 3.2.4. Need of Changing Nurses’ Attitudes towards Care 

According to the participants, nurses’ attitudes towards wound care may be improved significantly by being more motivated, striving towards quality improvement and developing an empathetic approach.

*“I think the solution for us is to become better, more motivated, more passionate with our work, more empathetic … if the nurse is not interested, nothing can be done …”*

(P1)

*“Another thing that is very important is to work with ourselves … on a behavioral level … it’s easy to lose your interest, to get angry, not to care … but that’s not the case. We should treat them (the patients) as if they were us, as if they were our relatives. I think that’s the key to the right thing to do.”*

(P7)

#### 3.2.5. Need of Changing the Patients’ Approach to Collaboration 

Approaching a patient to achieve collaborative care was a major concern for the participants. Despite the obstacles in caring for a mentally ill patients with skin injuries, nurses try to achieve the optimal level of collaboration with the patients. Patience, understanding and trusting relationships are issues that may enhance collaboration with patients.

*“We have to be patient, to talk to them (patients) calmly and steadily. If for example he (the patient) refuses to take care of his injury, we should explain to him why it is necessary to do so … and if he still refuses, another colleague should explain … until he is convinced.”*

(P5)

*“These patients need patience and understanding … and sometimes we are the only ones patients can rely on. Most patients here have no family, no one ever visits them … we are their caregiver, their family, their friends, everything … These people are in a very difficult position. That’s why we need to be understanding, trusting, and have a positive attitude towards them.”*

(P6)

## 4. Discussion

In the present qualitative study, through the views of the participating mental health nurses, the main factors that prevent the effective care of mentally ill patients with trauma are the shortage of hospital supplies and equipment, services and staff, the patient’s physical health and also the difficulty in collaborating with patients. As far as the nurses themselves were concerned, the lack of knowledge, the resistance they show to change, their beliefs and their emotional state were recognized as obstacles to care.

### 4.1. Factors Affecting the Care of Patients with Skin Injuries 

In the present study, the majority of participants reported that one important obstacle to wound care in a psychiatric hospital was the shortage of hospital supplies, equipment and services. This appeared to negatively affect nurses’ work and wound care. Similar results in the qualitative study of Woo et al. [16] highlighted the importance of using appropriate hospital supplies and equipment for the care of diabetic feet which, however, was not possible due to financial and managerial restrictions. Furthermore, Aletras and Kallianidou [17] found that the inappropriateness of hospital equipment is a significant obstacle to the performance of nurses, which is consistent with the findings of the present study. The same authors stressed that although in Greek hospitals managerial actions may often disregard the needs of health professionals, administrative support is necessary for overcoming barriers to nursing care. 

In the present study, shortages of staff emerged as an additional factor that influenced wound care. Similarly, Koukia and Gonis [18], in their study, identified staff shortages as one of the main clinical problems in providing care. Under-staffing leads to a reduction in the time of care provided to the patient, while at the same time limiting the possibility of effective intervention and inhibiting the therapeutic relationship with the patient. Respectively, in the ethnographic study of Cleary [19], the mental health nurses highlighted a lack of time as the main obstacle in the provision of care and expressed concerns about the growing demands on the workplace.

The present study found that the nurses’ lack of knowledge on wound care is a restrictive factor for wound healing. Similarly, Woo et al. [16] in their research demonstrated that physicians’ and nurses’ lack of knowledge about trauma was a deterrent to care, as they found that their practices did not comply with recommended guidelines and were therefore harmful to patients. In addition, Gallant et al. [20] who investigated nurses’ knowledge of pressure ulcers and follow-up practices found that the participants’ knowledge about the prevention and treatment of pressure ulcers was particularly insufficient. The discrepancy between the knowledge and clinical practice of nurses was particularly striking. Therefore, knowledge is essential for quality clinical practices and continuing education may contribute to its optimization [20]. 

In this study, several nurses expressed the view that many of their colleagues resisted change. This resistance resulted in utilization of invalid and outdated nursing practices. The same conclusion was reached by Ma et al. [21] who investigated the views of nurses on the factors that affect the application of knowledge in clinical practice after attending training programs. Nurses were reluctant to change due to the fear and uncertainty caused by the application of new practices and techniques. Finally, they expressed the belief that new practices require greater effort and workload and this was an additional reason for resisting change. 

Many participants in this study reported difficulties in collaborating with patients with mental illness and this impeded wound care severely. Cognitive stabilization, lack of motivation and compliance with care, inability of mental patients to accept nursing care or to respond to pain, fear to ask for help and denial were referred to as aggravating factors for the development of pressure ulcers [22,23]. Collaborating with mentally ill individuals was referred to as even more difficult when the patient was in an acute phase. Improvement of collaboration was possible when cognitive stabilization was reached, after medication [23]. An additional barrier to wound care is the patient’s aggressive behavior. Manifestations of aggressive behavior by patients are very common, as are incidents of violence against nurses. According to the relevant literature, patients’ aggressive behavior is one of the most difficult issues that nurses have to deal with in their workplace as it makes nurses work under fear and threat [24,25,26]. Baby et al. [27] stated that mental nurses perceived the patients’ aggressive behavior as a violation of their personal safety, which also followed them in their non-working life. 

In the present study, the patient’s physical condition was reported as an additional factor that affects the care of patients with skin injuries. The existence of comorbidities impedes wound healing. Khalil et al. [28] in their study refer to a plethora of comorbidities such as hypertension, diabetes, cardiovascular disease and musculoskeletal disorders that affected wound development and healing. In addition Athlin et al. [22] report that transfer of patients to other wards or hospitals due to other health problems results in discontinuity of care and becomes an additional risk factor for the development of pressure ulcers. 

According to the participants in this study, the lifestyle of the patients included factors such as malnutrition or obesity, prolonged bed rest and excessive smoking. Bhattacharya and Mishra [29] argue that malnutrition can cause significant delays in healing and accelerates the development of new ulcers. In addition, they confirm that patients with mental illness are at increased risk for developing pressure ulcers, as their diet is often poor in nutrients, resulting in hypoproteinemia. Similarly, the study of Black et al. [30] concluded that malnutrition alters tissue tolerance, inflammatory response and immunity status, making individuals more prone to ulcer development. The relevant literature demonstrates that excessive smoking is particularly common among patients with mental illness, while smoking prevents ulcer healing by affecting chemotaxis, migratory function and inflammatory response [31,32]. 

Many nurses in this study expressed views and beliefs that could deteriorate effective wound care. According to Baker [33], the attitudes of mental health nurses towards mentally ill patients negatively affect the quality of the provided nursing care and also the interactions between nurses and patients. The beliefs of nurses are greatly influenced by the stigma associated with mental illness which is spread by culture, society and professions. The Hamdan-Mansour and Wardam [34] study found that mental health nurses in Jordan had negative attitudes toward the mentally ill, describing them as dangerous, harmful, immature and emotionless. In addition, mental illness rated at a lower priority level than physical illness and verbal communication with mentally ill patients was considered useless. These beliefs may affect the quality of nursing care, and as a result, patients with mental illness may be deprived of adequate care.

The present study demonstrates that nurses’ years of employment affect their psychological status. Nurses who work many years with mentally ill patients experience feelings of frustration and anxiety, lack of motivation, lack of patience and anger. Similar results are reported in the study of Cleary [23], where nurses conveyed feelings of frustration especially when their interventions on mentally ill people failed. Furthermore, Ward [35] claimed that mental health nurses appeared to be anxious about working shifts, staff shortages and a chaotic and disorganized work environment. 

In addition, fatigue and exhaustion were reported to be experienced by nurses in the present study. According to the literature, acute psychiatric units are characterized by excessive workload, shortages of staff and a stressful working environment with unforeseen, unpredictable and aggressive incidents [35,36]. Often the health professionals are frustrated, questioning their personal abilities. This leads to apathy, lack of motivation and patience, irresponsibility, low self-esteem, feelings of guilt and defeatism [37,38].

### 4.2. Nurses’ Suggestions for Optimizing Care 

In the present study, the participants highlighted the need to employ more nurses and to recruit specialized nursing personnel in order to improve the care provided to mentally ill patients with skin injuries. Relevant studies report that adequate staffing may improve the quality of care as well as communication patterns with patients and therapeutic relationships [18,22]. In addition, nurses specialized in wound care have a strategic role in caring for patients with skin injures, assuring precision and efficacy of care and improving patient and staff satisfaction and safety in nursing practice [39,40].

Findings in this study highlighted that one of the main factors that impeded wound care was the lack of suitability and quantity of hospital supplies and equipment needed for wound care. Increasing the availability of hospital supplies and equipment could lead to optimized care. Similar findings are reported in the studies of Kalisch et al. [41] and Blackman et al. [42] according to which the second most important factor leading to inadequate nursing care is the lack of hospital supplies and equipment. Although many efforts have been made in order to increase the hospital supplies and the suitability of the materials and equipment, the problem still remains, leading to reduced productivity and development of side effects.

Another important issue that nurse participants revealed in the present study is the need of continuous and organized training on wound care. The lack of nurses’ knowledge appeared to be a restraining factor to quality of care and therefore educational interventions and in service training are suggested. Koukia and Gonis [18] stressed that mental health nurses are in need to update their knowledge and thus continuing education and reform of the existing educational programs are necessary. According to the study by Gallant et al. [20], training, administrative support and professional development are essential elements for optimizing care. 

Changing nurses’ attitudes towards wound care is referred to as an additional component for optimizing nursing care. More specifically, nurses focused on improving their professional status and developing empathy and motivation. These findings are in line with those reported in similar studies which underlined the importance of nurses to recognize their attitudes toward mental illness, to develop empathetic practices in caring and to focus on patient-centered care that supports patient recovery [43,44,45,46]. 

Furthermore, the participants in the present study highlighted the need to build a trusting relationship with the patient. In the same line, Cleary [23] argued that the nurse–patient relationship remains fundamental to the practice of nursing care. Developing a trusting relationship and having an empathetic and non-critical attitude towards patients were regarded as essential. Flexibility in nursing interventions, care with respect and dignity, patience and confidentiality were also reported as major features of optimal care.

Confronting effectively the patients’ aggressive behavior was also associated with the provision of optimal care. In the study of Lantta et al. [47], participants conveyed many suggestions on how to prevent aggressiveness effectively. These suggestions are fit into four main categories: in-service training, effective interaction, adequate staffing and improved safety. More specifically, high quality in-service training for nursing staff may lead to better treatment, effective and consistent care, safe practices and accurate monitoring. 

### 4.3. Limitations of the Study

The main limitation of this study was the small number of available participants, as the health care services for patients with mental illness are usually difficult to contact. In addition, this study was one of the few studies that examined the views of nurses on the mental care of patients with skin injuries at national and international level, so the existing literature was very limited.

### 4.4. Trustworthiness of Research

In order to improve credibility and establish trustworthiness the researcher had prolonged engagement by investing sufficient time in the data collection activities to learn the culture of the group under study, to test for misinformation and distortions and to build trust with informants. For this purpose the researcher-interviewer in this study was involved in the area of investigation long before the data collection commencement by visiting the mental health hospital, being introduced to nurses and talking to them. Reflexivity, research triangulation, member check and thick description are some of the strategies that have been used to establish trustworthiness of the study. 

## 5. Conclusions

Participants provided a range of suggestions that would help optimize the care provided. These proposals focused on the need for additional staff, the need for increasing hospital supplies and equipment availability and the need for updating nurses’ knowledge. In addition, they stressed the need to change nurses’ attitudes toward care, but also the need to change the approach of patients, in order to achieve collaboration. 

In conclusion, according to nurses, the factors that affect the care of mentally ill patients with skin injuries are multidimensional and the effort to remove them, with the aim of achieving effective care, is considered imperative.

### Expected Benefits from the Study

This study may help to better understand the views of nurses who care for patients with mental illness and skin injuries, thus generating new knowledge for the scientific community. In addition, its contribution could be particularly important in improving the care provided to patients with skin injuries, both in mental health or general hospitals.

In conclusion, the results of the study could be used to develop action plans and continuing education programs, either by nurses or by the respective administration. Finally, the results of the research can give rise to new studies on the subject under study, with the aim of a deeper understanding.

## Figures and Tables

**Table 1:** Demographic data of study participants.

Participants’ No	Gender	Age	Education	Years of Employment in Psychiatric Ward
P1	M	45	MSc	14
P2	M	41	MSc	7
P3	F	42	BSc	22
P4	F	44	BSc	21
P5	F	26	BSc	1
P6	F	43	BSc	15
P7	F	39	MSc	10

**Table 2:** Main themes and subthemes.

Main Themes	Factors Affecting the Care of Patients with Skin Injuries	Nurses’ Suggestions for Optimizing Care
Subthemes	(a) The shortage of hospital supplies, equipment and services	(a) Need for additional staff
	(b) The shortage of staff	(b) Need for increasing hospital supplies and equipment availability
	(c) The lack of knowledge	(c) Need for training
	(d) The nurses’ resistance to change	(d) Need of changing nurses’ attitudes towards care
	(e) The difficulty in collaborating with patients	(e) Need of changing the patients’ approach to collaboration
	(f) The patients’ physical condition	
	(g) The nurses’ attitudes as an obstacle to care	
